# Beautiful Samples

**Published:** 1884-01-20

**Authors:** 


*Beautiful Samples.*—Among the most beautiful samples of fine pharmaceutical preparations we have ever seen are those left at our office by a member of the house of Fairchild Brothers & Foster, of New York. We propose to test these preparations in practice, as we are very favorably impressed with their appearance and with the gentleman who kindly left them with us.

